# Clinical Presentations, Antiplatelet Strategies and Prognosis of Patients with Stent Thrombosis: An Observational Study of 140 Patients

**DOI:** 10.1371/journal.pone.0048520

**Published:** 2012-10-31

**Authors:** Ya-Ling Han, Quan-Yu Zhang, Yi Li, Shao-Yi Guan, Quan-Min Jing, Zu-Lu Wang, Xin Zhao, Xiao-Zeng Wang, Ying-Yan Ma, Bin Wang, Jie Deng, Geng Wang, Young-Hak Kim

**Affiliations:** 1 Department of Cardiology, Shenyang Northern Hospital, Shenyang, Liaoning, China; 2 Cardiology Department, Asan Medical Center, University of Ulsan College of Medicine, Seoul, Korea; University Medical Center Utrecht, The Netherlands

## Abstract

**Background:**

Until now there has been scarce evidence regarding an optimal antiplatelet strategy and clinical outcomes for patients who had suffered from stent thrombosis (ST).

**Methods and Results:**

140 patients who suffered from stent thrombosis were prospectively registered. Patients received dual (aspirin and 150 mg clopidogrel, N = 66) or triple (additional cilostazol, N = 74) antiplatelet therapy at the physician’s discretion. Thereafter platelet reactivity and one year clinical outcomes were analyzed. The primary outcome included the composite of cardiac death, non-fatal myocardial infarction (MI) or stroke at one year,which developed in 41 (29.3%) patients, consisting of 31 (22.1%) cardiac death, 9 (6.4%) non-fatal MI and 1 (1.4%) stroke. Recurrent definite and probable ST according to ARC definition was observed in 8 (5.7%) and 14 (10.0%) patients, respectively. Triple therapy was associated with significantly lower platelet reactivities (50.2±17.8, % vs. 59.6±17.2, %, *P* = 0.002) compared to high dose dual antiplatelet therapy. However, the incidence of primary events (24.3% vs. 34.8%, *P* = 0.172) did not differ between triple and dual antiplatelet therapies. High on-treatment platelet reactivity (HR: 8.35, 95% CI: 2.234∼30.867, *P* = 0.002) and diabetes (HR: 3.732, 95% CI: 1.353∼10.298, *P* = 0.011) were independent predictors of primary events.

**Conclusions:**

Patients who suffered from stent thrombosis have a poor prognosis even after revascularization with intensive antiplatelet therapy. Triple antiplatelet therapy was more effective in reducing on-treatment platelet reactivity, compared to high dose dual antiplatelet therapy.

## Introduction

Stent thrombosis (ST) is a life-threaten complication for patients with coronary artery stent implantation. Although dual antiplatelet treatment with aspirin and clopidogrel has been recommended as the standard therapy for the prevention of thrombotic events in patients undergoing percutaneous coronary intervention (PCI), the incidence of ST persists at a rate of 0.5% to 2% [1]–[3]. Therefore, higher clopidogrel loading and maintenance doses, as well as the administering of additional cilostazol, which exerts a more potent antiplatelet effect, have been employed in some prospective studies and were reported effective in reducing long-term adverse clinical events after PCI in selected patients [4], [5]. Conditions of patients who already had ST episodes were usually more complicated and more critical than patients who did not have ST after PCI. But the preferred pharmacal therapy strategy, and the long-term relation between treatment options and platelet reactivity for ST patients has not been proved. Therefore, the purpose of the present study is to explore the optimal antiplatelet strategies and prognostic predictors for patients who already had ST.

## Methods

### Patient Population

**Figure 1:**
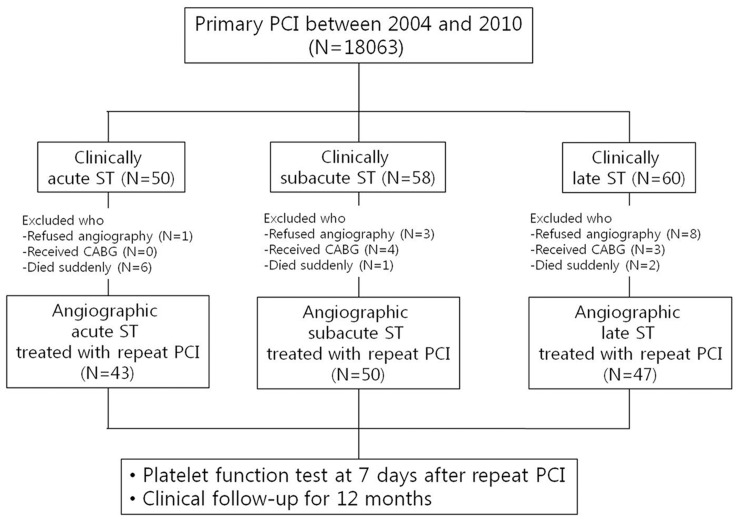
Study flow chart. CABG, coronary artery bypass graft surgery; ST, stent thrombosis; PCI, Percutaneous coronary intervention.

From Jan 2004 to Mar 2010, a total of 140 patients with angiographically confirmed ST were prospectively registered in this study. The angiographic criteria of stent thrombosis consisted of partial or complete occlusion within the previously implanted stents with evidence of fresh thrombus. Excluded from the study were patients who were suspected to have ST but refused to receive coronary angiography or percutaneous revascularization, patients undergoing emergent surgical revascularization and patients who did not survive the emergent PCI (Figure 1). The study was approved by the hospital ethics committee. All patients gave their informed consent.

### Coronary Revascularization and Medications

All eligible patients received coronary balloon angioplasty and/or stent implantation in the affected vessel after emergent angiography. Then, antiplatelet therapies were adjusted at the physicians’ discretion. Among them, 66 patients received high dose dual antiplatelet therapy (300 mg of aspirin per day for 30 days followed by 100 mg per day indefinitely, 150 mg of clopidogrel per day for 30 days followed by 75 mg per day for at least 1 year), and 74 patients received triple antiplatelet therapy (300 mg of aspirin per day for 30 days followed by 100 mg per day indefinitely, 75 mg of clopidogrel for at least 1 year and 100 mg of cilostazol [Otsuka Pharmaceutical Co., Ltd. Japan] twice a day for 6 months). Platelet glycoprotein IIb/IIIa receptor inhibitors (GPI) were used in all eligible patients for 12∼48 hours since its availability at our center from 2006. Cardiovascular medications, such as heparin/low molecular weight heparin, statins, beta-blockers, angiotensin-converting enzyme inhibitors, and calcium antagonists were used according to contemporary guidelines.

### Assessment of Platelet Aggregation (PA)

Blood samples were collected in the cath lab before emergent coronary angiography and at 7 days after the adjustment of antiplatelet therapy or at the onset of primary events. PA was assessed by light transmission aggregometry. Blood was centrifuged (200 g×10 min) to obtain platelet-rich plasma. The platelet count of the platelet-rich plasma (PRP) was adjusted to a range of 150,000 to 300,000 platelets/L by dilution with autologous plasma when not within range. The remaining specimen was re-centrifuged (1,500 g×15 min) to obtain platelet-poor plasma. Platelets were stimulated with 20 µmol/L ADP. Aggregation was measured at 37°C with a PACKS-4 or AggRAM-TMA (Helena Laboratories, Beaumont, Texas) and expressed as the maximal percentage change in light transmittance from the baseline to 5 min after the addition of the agonist, using the platelet-poor plasma (PPP) as a reference.

### Definitions of Clinical Events

The primary outcome was the occurrence of ischemic events [6], and defined as one of cardiac death, non-fatal myocardial infarction (MI), or a stroke at 12 months. Secondary outcomes included recurrent ST (re-ST, i.e. second time ST), bleeding, and all separate components of primary outcome. Non-fatal MI included reinfarction (defined as the recurrence of symptoms with ST-segment elevation or a new left bundle branch block and an increase in cardiac enzymes after stable or decreasing values) or spontaneous MI (diagnosed by a 300% rise in creatine kinase-MB along with symptoms and either the development of ST-segment elevation or a new left bundle branch block) according to American College of Cardiology/American Heart Association Guidelines [7]. Stroke was defined as an ischemic cerebral infarction caused by the embolic or thrombotic occlusion of a major intracranial artery. Re-ST was classified as definite, probable, or possible, according to definitions proposed by the Academic Research Consortium, ^8^ and it was stratified as acute (≤24 h), subacute (24 h to 30 days), and late (30 days to 1 year). Bleeding events were classified according to Thrombolysis In Myocardial Infarction criteria.^9^ Major bleeding was defined as any intracranial bleeding or any bleeding associated with clinically overt signs and a drop in hemoglobin >5 g/dl. Minor bleeding was defined as any clinically overt sign of bleeding associated with a 3–5 g/dl decrease in hemoglobin. Minimal bleeding was defined as any clinically overt sign of hemorrhage (including imaging) that is associated with a <3 g/dl decrease in the hemoglobin concentration or <9% decrease in the hematocrit. Patient medical records were reviewed to substantiate recorded events. Clinical follow-up was performed by telephone or outpatient visits at 30 days, 6 months, and 12 months after enrollment. All adverse events, drug compliance, and side effects were recorded in our follow-up database.

### Statistical Analysis

Continuous variables are presented as mean ± standard deviation (SD), and categorical variables are expressed numerically and/or as percentages. Categorical variables were compared by Pearson chi-square test or by Fisher’s exact test, and continuous variables were compared by means of Student’s t test. To evaluate whether PA assay could be an effective predictor with or without a primary end point at a 1-year follow-up, a receiver operating characteristic (ROC) curve analysis was performed. The optimal cutoff level was calculated by determining the shortest distance between the ROC curve and the upper left corner of the graph. Patients above the optimal cutoff level were considered to exhibit high on-treatment platelet reactivity (HPR). Survival curves were calculated by Kaplan-Meier models. Independent predictors were calculated by Cox regression models. All available variables considered potentially relevant were: age, gender, hypertension, diabetes mellitus, smoking, family history, cerebrovascular disease, hyperlipidemia, nephropathy, multivessel disease, multi-stenting, stent overlapping, number of lesions treated, number of stents implanted, average stent diameter, total length of stents, PA function, and concomitant medications. Results are presented as adjusted hazard ratio (HR) with 95% confidence interval (CI). All statistical tests were 2-tailed, and a P value ≤0.05 was considered statistically significant. Statistical analyses were performed with SPSS 16.0 software (SPSS Institute, Chicago, Illinois).

## Results

### Baseline Characteristics

Baseline details were shown in Table 1 and Table 2. The average age in the cohort was 63.3±11 years. Risk factors of all the patients were shown as below: 49.3% patients were smokers. 25.7% patients had prior myocardial infarctions. 30.7% of patients suffered from diabetes. 14.3% patients had prior stroke. Three patients had renal dysfunction and two patients had Peripheral arterial disease. Medications of all the patients were presented as below: All patients take aspirin and clopidogrel(Oral Tablets). 71.4% patients take cilostazol. Usage rate of statins, β blocker and ACEI were 62%, 70.7%, 32.1, respectively. Average Serum Creatinine, hs-CRP, platelet count, and PA were 97.5±24.9 µmol/L, 5.9±16 ng/L, 219.3±89.9 109/L, 71.8±8.6%, respectively. The time distribution of stent thrombosis was acute in 43 (30.7%), subacute in 50 (35.7%), and late in 47 (33.6%) patients. All 126 patients (90%) received stent implantation. GPI was administered in 73 patients (52.1%) in the later study period since 2006.

**Table 1:** Baseline clinical characteristics.

Variables	N = 140
Age(years)	63.3±11.0
Male, %	111(79.3)
Risk factors	
Smoking	69(49.3)
Prior myocardial infarction	36(25.7)
Hypertension	72(51.4)
Diabetes mellitus	43(30.7)
Renal dysfunction	3(2.1)
Prior stroke	20(14.3)
Peripheral arterial disease	2(1.4)
Medications at admission	
Aspirin	140 (100)
Clopidogrel	140 (100)
Cilostazol	74 (52.9)
Statins	87(62.0)
β blocker	99(70.7)
ACEI	45(32.1)
Laboratory determinations	
Serum Creatinine, μ mol/L	97.5±24.9
TnT positive	137(97.9)
hs-CRP, ng/L	5.9±16
Platelet count, 10^9^/L	219.3±89.9
Platelet aggregation(%)	71.8±8.6
Type of stent thrombosis	
Acute (≤24 hours)	43(30.7)
Subacute (1–30 days)	50(35.7)
Late (30 days-1 year)	47(33.6)

Values were presented as number (%) and mean±SD. ACEI, Angiotensin converting enzyme inhibitor; TnT, troponin T, CRP, C-reactive protein.

**Table 2:** Baseline angiographic and PCI results.

Variables	N = 140
Multivessel disease	63(45)
Previous PCI	
Stent diameter, mm	2.9±0.71
Total stent length, mm	47.7±26.5
Number of stents	2.0±1.3
Bifurcation stenting	79(56.4)
Stent overlapping	35(25)
DES implantation	84(60)
Revascularization strategy	
Stent implantation	126(90)
Balloon dilatation	14(10)
Antithrombotic strategies	
IIb/IIIa receptor inhibitor	73(52.1)
Triple antiplatelet	74(52.9)
High dose dual antiplatelet	66(47.1)
Heparin/LMWH	87(62.1)

Values were presented as number (%) and mean±SD. PCI, percutaneous coronary intervention, LMWH, low molecular weight heparin.

### One-year Clinical Outcomes

**Figure 2:**
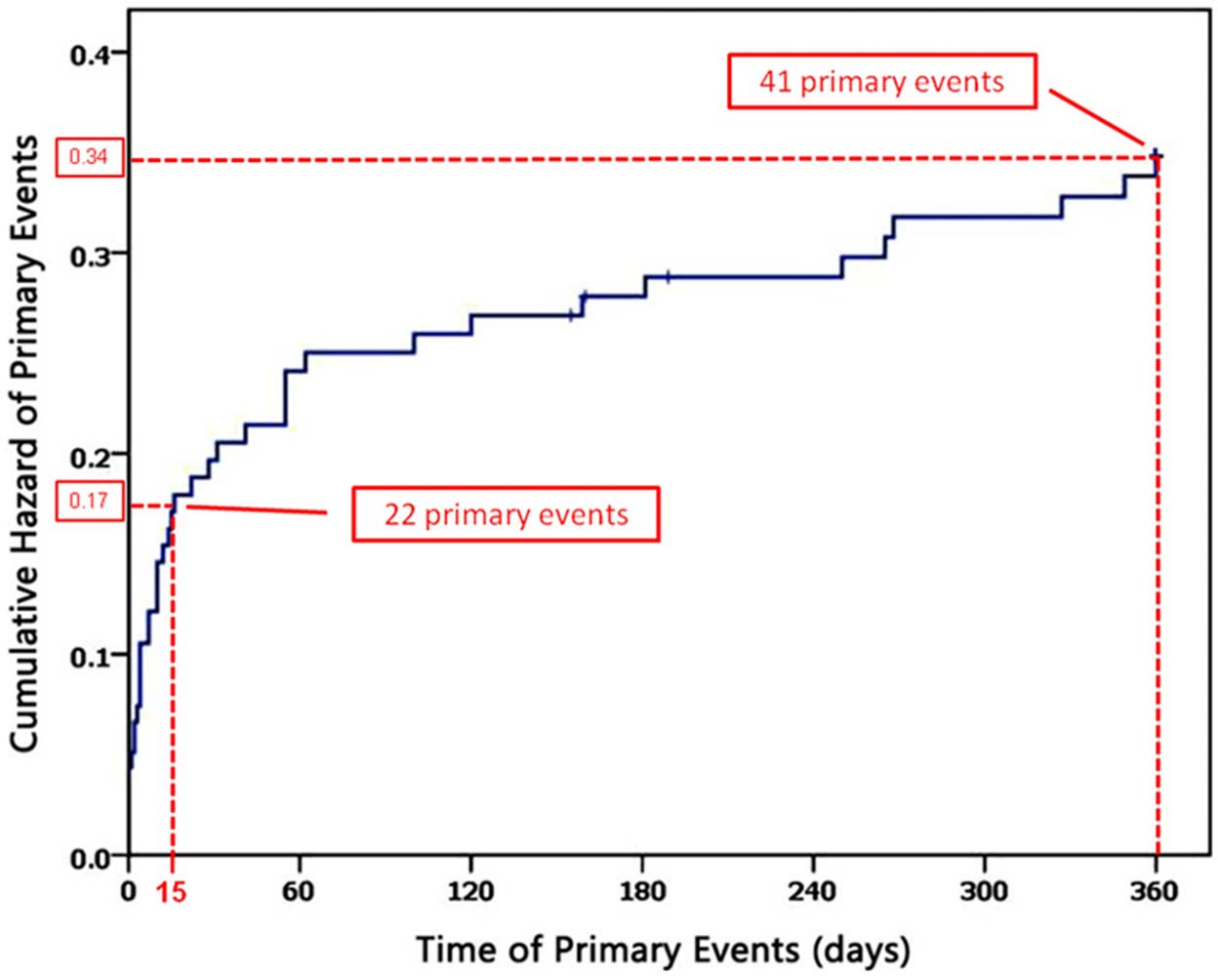
Time distribution of primary events. Blue curve indicated cumulative hazard of primary events. Before the 15^th^ day, 22 primary events developed, cumulative hazard was 0.17, number of total primary events was 41 in one year, cumulative hazard was 0.34.

One-year clinical outcomes are described in Table 3. At the one-year follow-up, primary events developed in 41 (29.3%) patients, which contained 31 (22.1%) cardiac death, 9 (6.4%) non-fatal MI, and 1 (1.4%) stroke. Recurrent definite (N = 8) or probable (N = 14) ST according to the ARC definition was observed in 22 (15.7%) patients within one year. One year repeat revascularization rate was 19.3% and was not different between the triple (17.6%) and dual (21.2%, p = 0.59) antiplatelet groups. The Kaplan-Meier curve showed that approximately 50% of all primary events developed in the first 15 days (Figure 2).

**Table 3:** Table **3.** Clinical outcomes at 1 year.

Clinical event	N = 140
Primary events	41(29.3)
Cardiac death	31(22.1)
Non-fatal myocardial infarction	9(6.4)
Stroke	2(1.4)
Recurrent stent thrombosis	37(26.4)
Definite	8(5.7)
Probable	14(10)
Possible	15(10.7)
Repeat revascularization	27(19.3)
Target vessel	27(19.3)
Non-target vessel	0(0)
TIMI bleeding events	13(9.3)
Major	2(1.4)
Minor	0(0)
Minimal	11(7.9)

All values were presented as number and actual incidence.

### Triple Versus High Dose Dual Antiplatelet Therapy

**Figure 3:**
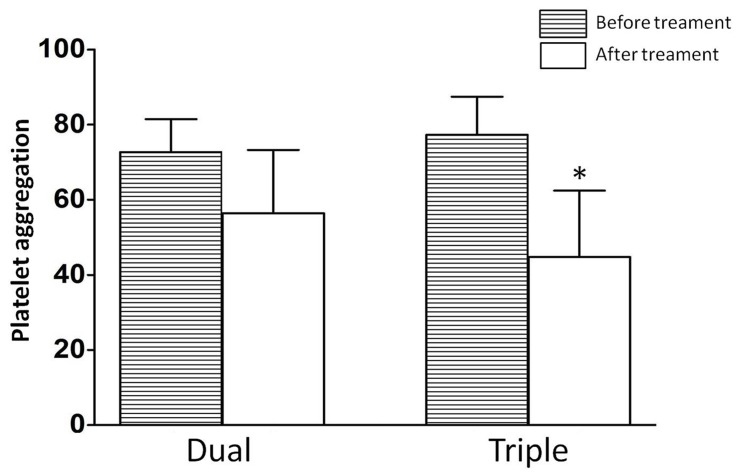
Changes of PA in dual and triple antiplatelet therapy. Before treatment PA was performed before clinical treatment, after treatment PA was performed after 7 days under intensive therapy (^*^Triple-After treatment vs. Dual-After treatment, P = 0.002).

Baseline characteristics and laboratory analyses of patients receiving the two different antiplatelet regimens are shown in Table 4. There were no significant differences between the two groups according to age, gender, risk factors, and medications, with the exception that patients in the dual therapy group had a higher proportion of heparin treatment compared with patients in the triple therapy group (80.6% vs. 42.6%, P<0.001). Baseline PA assay results were similar between the two groups (71.1±9.2% vs. 72.7±7.9%, P = 0.260). A significant reduction in PA was observed in both groups after adjusting for antiplatelet therapies. Patients receiving triple antiplatelet therapy achieved a stronger PA inhibition than those receiving high dose dual antiplatelet therapy (50.2±17.8% vs. 59.6±17.2%, P = 0.002, Figure 3). Triple antiplatelet therapy was associated with a non-significant 30.1% relative risk reduction on primary events (24.3% vs. 34.8%, P = 0.172). The cumulative incidences of recurrent definite or probable ST (10.8% vs. 21.1%, P = 0.091) were not significantly different between the two groups but numerically lower for patients receiving triple antiplatelet therapy.

**Table 4:** Baseline characteristics and laboratory determinations for patients with dual or triple antiplatelet therapy.

Variables	Dual therapy, n = 66	Triple therapy, n = 74	P value
Age (years)	64.3±10.7	62.3±11.3	0.284
Male	50 (75)	62 (83.8)	0.198
Risk factors			
Smoking	28 (41.7)	42 (57.4)	0.064
Old myocardial infarction	16 (23.6)	21 (27.9)	0.558
Hypertension	30 (45.8)	42 (57.4)	0.173
Diabetes	23 (34.7)	20 (26.5)	0.29
Kidney disease	2(2.8)	1 (1.5)	0.593
Stroke	6 (9.7)	14 (19.1)	0.112
Peripheral vascular disease	2 (2.8)	0	0.166
Stent diameter, mm	2.8±0.9	2.9±0.5	0.222
Stent length, mm	62.5±24.6	65.1±27.5	0.557
Number of stents	1.8±1.3	2.1±1.3	0.214
Multivessel disease	28 (41.7)	36 (48.5)	0.415
Stent implantation	57 (86.4)	69 (93.2)	0.176
Medications			
Heparin	53 (80.6)	32 (42.6)	<0.001
Statins	46 (69.4)	40 (54.4)	0.067
β blocker	42 (63.9)	58 (77.9)	0.068
IIb/IIIa inhibitor	30 (45.5)	43 (58.1)	0.135
Laboratory determinations			
Serum Creatinine (µmol/L)	97.5±24.0	96.9±26.0	0.871
Platelet count (/L)	222.1±101.5	216.4±76.5	0.708
CK (U/L)	431.3±900.6	357.5±905.9	0.63

Values were presented as number (%) and mean±SD. CK, creatine kinase.

### Predictive Values of on-treatment Platelet Reactivity on Prognosis

**Figure 4:**
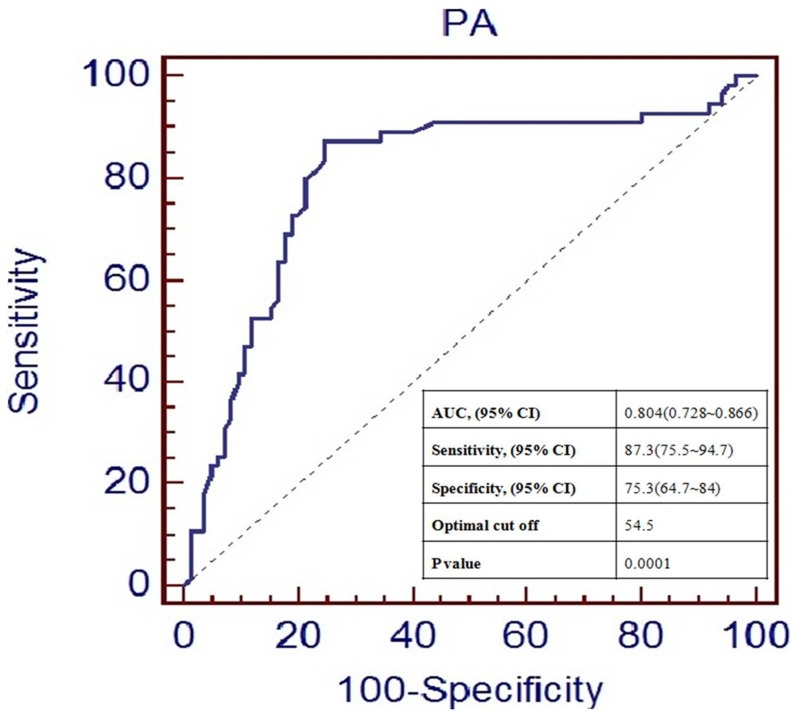
ROC curve analysis in PA and ischemic events. PA, platelet aggregation; AUC, area under the curve; CI, confidence interval. Cut off value was calculated by determining the smallest distance between receiver operating characteristic curve and upper left corner of the graph.

**Figure 5:**
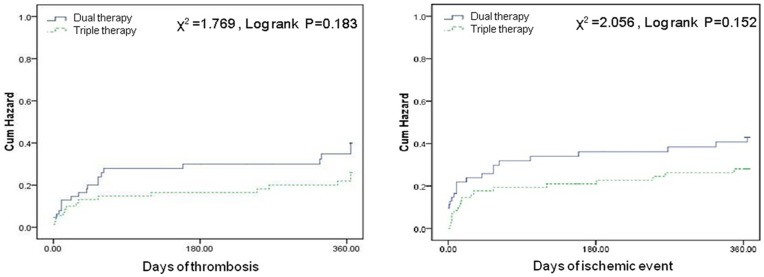
Kaplan-Meier curves of cumulative hazard of recurrent stent thrombosis (left) and ischemic events (right) of patients received high dose dual or triple antiplatelet therapy.

ROC curve analysis demonstrated that PA assay was able to distinguish between patients with and without ischemic events at the one-year follow-up (Figure 4). The area under the curve (AUC) was 0.804 and the optimal cutoff value for PA was 54.5%. Patients without HPR had lower incidences of primary events (12.3% vs. 44%, P<0.001) and recurrent definite or probably ST (4.6% vs. 25.3%, P = 0.001) compared with patients with HPR (Figure 5).

### Prognostic Predictors

Multivariate analysis showed that HPR (HR: 8.35, 95% CI: 2.234∼30.867, p = 0.002) and diabetes (HR: 3.732, 95% CI: 1.353∼10.298, p = 0.011) were independent predictors of primary events. Triple antiplatelet strategy was not significantly associated with primary events (HR: 0.650; 95% CI: 0.187∼2.256, P = 0.497).

## Discussion

To our knowledge, the present study was the first study with the largest sample size to evaluate antiplatelet therapy strategy and prognosis in patients with ST in Asia. The principal findings of the present study were as follows: first, patients with ST had high incidences of major adverse clinical events (29.3%) and re-ST (15.7%, see table 3) even after urgent coronary revascularization and intensive antiplatelet therapy. Second, a high on-treatment platelet aggregation rate is effective in predicting adverse clinical events in patients with ST. Third, triple antiplatelet therapy with cilostazol on top of aspirin and clopidogrel achieved stronger platelet aggregation inhibition compared with high dose dual antiplatelet therapy. But clinical event rate did not differ. So we need a further research.

With the recent progress of devices and techniques, PCI has become one of the safest and most effective therapies for coronary artery disease. Previous studies have reported that one-year cumulative incidences of cardiac death (about 0.5%) and ST (0.5% ∼2%) after coronary stenting in real-world PCI practice [1], [8], [10], [11]. But the clinical consequences of angiographically confirmed ST were very severe, resulting in high rates of mortality (20%∼40%) and morbidity, including nonfatal MI in 70% of cases [1], [8], [12]–[15]. Lots of studies reported the incidences and predictors of first ST episodes [16], [17]. Recently, Yeo KK, et al published their study [18] on in-hospital clinical outcomes and characteristics of 153 angiographically confirmed ST patients, but the outcomes of long term antiplatlet regimen after the first ST episodes has not been reported until now. While from the present study, for the first time, we found the one year clinical outcomes of re-ST for patients who already suffered from ST episodes, predictors for the re-ST and the relationship between re-ST and PA test. The incidences of cardiac death and re-ST in patients at the one-year follow-up were 22.1% and 15.7%, respectively, which were about 8 folds higher than that for regular PCI patients even after urgent revascularization and intensified antiplatelet treatment. The reasons for such a poor prognosis may be multi-factorial. As shown in the baseline data of present study, the high proportion of low response to antiplatelet treatment and diabetes, which were consistent with previously reported as independent predictors of ST[19∼21], may play important roles in re-ST and major ischemic events. Moreover, extended myocardial damage caused by two or more times of ST related infarction and secondary mental depression may make patients more vulnerable. Another significant finding of the present study was that a cluster of early major events occurred within the first 15 days after urgent PCI for treatment of first-time ST, which indicates the importance of early intensive care and monitoring for patients with ST.

HPR is a proven predictor of ischemic events. However, its role in predicting re-ST for patients receiving intensive antiplatelet treatment has not been well elucidated. ROC curve analysis in present study demonstrated a relationship between PA and ischemic events, which is consistent with the POPULAR study [22]. Meanwhile, patients with HPR, tested on 7^th^ days after ST and categorized by optimal cutoff value, had poor clinical prognoses compared with patients with normal on-treatment platelet reactivity. The present study also demonstrated that HPR was an independent predictor of major ischemic events for patients with ST, which were not reported by previous studies in such cohort. Based on p revious studies, HPR is not merely a laboratory indication of responsiveness to antiplatelet treatment but is also closely associated with adverse clinical events [23∼26,12]. Therefore, it is reasonable to consider it as an effective predictor of adverse ischemic events in patients with ST.

Since scarce evidence up to now, there has been no consensus on the optimal pharmacal therapy regarding the antithrombotic regimen for patients already suffering from intracoronary ST. As most of the thrombotic events were mainly caused by a poor response to standard antiplatelet treatment [13], adjusted regimens such as an increase in the clopidogrel dose or combining antiplatelet agents with different medications were studied to attenuate antiplatelet resistance. Present study suggested that triple therapy with cilostazol on top of routine dosages of aspirin and clopidogrel had stronger inhibition of platelet aggregation compared to dual antiplatelet treatment with high dose clopidogrel for patients already suffering from intracoronary ST, which was consistent with the finding of previous studies which treated for first-time ST [14], [15], [27]. Furthermore, DECREASE registry found that triple antiplatelet therapy(standard antiplatelet treatment combined with cilostazol) reduced first episode ST after drug-eluting stent implantation [28]. However, the clinical outcome for preventing re-ST by the triple antiplatelet regimen was unclear. The present study initially explored the potential benefit of triple antiplatelet regimen on long term clinical outcomes. Although there was no significant difference between the triple and dual groups, a trend of 30.1% of relative risk reduction has been found, which may be attributed to the inadequate sample size and generates a hypothesis for further prospective large-scale clinical studies.

### Limitations

The present study was an observational study with its inherent shortcomings. First, because of non-randomized study design, our finding on the optimal antiplatelet regimen should be considered as hypothesis-generating. Although this has been one of the largest studies enrolling patients with ST, the sample size may still be underpowered to determine the clinical advantages of various antiplatelet regimens. Second, we included ST occurring in the early as well as late periods after procedure. But the mechanisms of ST and prognostic factors may not be homogenous in each study period. Third, because our study only included patients who presented with ST and received repeat PCI, which did not represent the entire profile of ST patients as severe acute ST patients usually had no chance and enough time to receive PCI procedure. Forth, the treatment selection of ST was left to the physicians. Therefore, the diverse treatment strategies might have contributed to the outcome. Fifth, this study analyzed the outcomes in an intention-to-treat principle. Therefore, crossover between antiplatelet regimens might have diluted the real benefit of triple therapy. Besides, we did not systemically capture the information on drug compliance and impact of drug-crossover could not be assessed.

In conclusion: First, patients with ST have a poor prognosis even after revascularization with intensive antiplatelet therapy. Second, HPR was an independent predictor of adverse cardiac events for patients with ST. Third, triple antiplatelet therapy was associated with more potent platelet inhibition and a non-significant 30.1% relative risk reduction on adverse clinical events compared to dual antiplatelet therapy with high dose clopidogrel. Further researches are warranted to assess the role of platelet function test and identify the optimal antiplatelet regimen for patients with ST.

## References

[1] Cutlip DE, Baim DS, Ho KK, Popma JJ, Lansky AJ, et al (2001) Stent thrombosis in the modernera: a pooled analysis of multicenter coronary stent clinical trials. Circulation 103: 1967–1971. 11306525 10.1161/01.cir.103.15.1967

[2] Orford JL, Lennon R, Melby S, Fasseas P, Bell MR, et al (2002) Frequency and correlates of coronary stent thrombosis in the modern era: analysis of a single center registry. J Am Coll Cardiol 40: 1567–1572. 12427407 10.1016/s0735-1097(02)02374-4

[3] Takayama T, Hiro T, Hirayama A (2011) Stent thrombosis and drug-eluting stents. J Cardiol 58: 92–98. 21839615 10.1016/j.jjcc.2011.07.003

[4] Han Y, Li Y, Wang S, Jing Q, Wang Z, et al (2009) Cilostazol in addition to aspirin and clopidogrel improves long-term outcomes after percutaneous coronary intervention in patients with acute coronary syndromes: a randomized, controlled study. Am Heart J 157: 733–739. 19332203 10.1016/j.ahj.2009.01.006

[5] Chen KY, Rha SW, Li YJ, Poddar KL, Jin Z, et al (2009) Triple versus dual antiplatelet therapy in patients with acute ST-segment elevation myocardial infarction undergoing primary percutaneous coronary intervention. Circulation 119: 3207–3214. 19528339 10.1161/CIRCULATIONAHA.108.822791

[6] Fei Gao, Yu Jie Zhou, Zhi Jian Wang, Hua Shen, Xiao Li Liu, et al (2010) Comparison of Different Antithrombotic Regimens for Patients With Atrial Fibrillation Undergoing Drug-Eluting Stent Implantation. Circulation Journal 74: 701–708. 20208381 10.1253/circj.cj-09-0880

[7] Ryan TJ, Faxon DP, Gunnar RM, Kennedy JW, King SB 3rd, et al (1988) Guidelines for percutaneous transluminal coronary angioplasty. A report of the American College of Cardiology/American Heart Association Task Force on Assessment of Diagnostic and Therapeutic Cardiovascular Procedures (Subcommittee on Percutaneous Transluminal Coronary Angioplasty). J Am Coll Cardiol 12: 529–545. 2969021

[8] Ong AT, Hoye A, Aoki J, van Mieghem CA, Rodriguez Granillo GA, et al (2005) Thirty-day incidence and 6-month clinical outcome of thrombotic stent occlusion after bare-metal, sirolimus, or paclitaxel stent implantation. J Am Coll Cardiol 45: 947–953. 15766834 10.1016/j.jacc.2004.09.079

[9] Rao AK, Pratt C, Berke A, Jaffe A, Ockene I, et al (1988) Thrombolysis In Myocardial Infarction (TIMI) Trial. Phase I: Hemorrhagic manifestations and changes in plasma fibrinogen and the fibrinolytic system in patients treated with recombinant tissue plasminogen activator and streptokinase. J Am Coll Cardiol 1988 11: 1–11. 10.1016/0735-1097(88)90158-1 3121710

[10] Li Y, Li CX, Wang HC, Xu B, Fang WY, et al (2011) Efficacy and safety of Firebird sirolimus-eluting stent in treatment of complex coronary lesions in Chinese patients: one-year clinical and eight-month angiographic outcomes from the FIREMAN registry. Chin Med J (Engl) 124: 817–824. 21518586

[11] Garg S, Serruys PW, Silber S, Wykrzykowska J, van Geuns RJ, et al (2011) The prognostic utility of the SYNTAX score on 1-year outcomes after revascularization with zotarolimus- and everolimus-eluting stents: a substudy of the RESOLUTE All Comers Trial. JACC Cardiovasc Interv 4: 432–441. 21511223 10.1016/j.jcin.2011.01.008

[12] Geisler T, Grass D, Bigalke B, Stellos K, Drosch T, et al (2008) The Residual Platelet Aggregation after Deployment of Intracoronary Stent (PREDICT) score. J Thromb Haemost 6: 54–61. 17949474 10.1111/j.1538-7836.2007.02812.x

[13] Elsenberg EH, van Werkum JW, van de Wal RM, Zomer AC, Bouman HJ, et al (2009) The influence of clinical characteristics, laboratory and inflammatory markers on ‘high on treatment platelet reactivity’ asmeasured with different platelet function tests. Thromb Haemost 102: 719–727. 19806258 10.1160/TH09-05-0285

[14] Kereiakes DJ, Choo JK, Young JJ, Broderick TM (2004) Thrombosis and drug-eluting stents: a critical appraisal. Rev Cardiovasc Med 5: 9–15. 15029110

[15] Popovic B, Casu AG, Angioi M, Moulin F, Piquemal R, et al (2005) Acute or sub-acute thrombosis of steel stents. Arch Mal Coeur Vaiss 98: 1187–1191. 16435596

[16] Lemesle G, Delhaye C, Bonello L, de Labriolle A, Waksman R, et al (2008) Stent thrombosis in 2008: definition, predictors, prognosis and treatment. Arch Cardiovasc Dis 101: 769–777. 19059572 10.1016/j.acvd.2008.10.006

[17] Trabattoni D, Fabbiocchi F, Montorsi P, Ravagnani P, Galli S, et al (2007) Stent thrombosis after sirolimus- and paclitaxel-eluting stent implantation in daily clinical practice: analysis of a single center registry. Catheter Cardiovasc Interv 70: 415–421. 17722020 10.1002/ccd.21149

[18] Yeo KK, Mahmud E, Armstrong EJ, Bennett WE, Shunk KA, et al. (2011) Contemporary clinical characteristics, treatment, and outcomes of angiographically confirmed coronary stent thrombosis: Results from a multicenter California registry. Catheter Cardiovasc Interv. *In press*. 10.1002/ccd.23011 21563289

[19] Gurbel PA, Bliden KP, Samara W, Yoho JA, Hayes K, et al (2005) Clopidogrel effect on platelet reactivity in patients with stent thrombosis: results of the CREST study. J Am Coll Cardiol 46: 1827–1832. 16286166 10.1016/j.jacc.2005.07.056

[20] Davì G, Vazzana N, Sestili S. (2012) Variability in the response to antiplatelet treatment in diabetes mellitus. Prostaglandins Other Lipid Mediat. *In press*. 10.1016/j.prostaglandins.2012.01.008 22330860

[21] Iakovou I, Schmidt T, Bonizzoni E, Ge L, Sangiorgi GM, et al (2005) Incidence, predictors, and outcome of thrombosis after successful implantation of drug eluting stents. JAMA 293: 2126–2130. 15870416 10.1001/jama.293.17.2126

[22] Pramod K, Kuchulakanti, Chu WW, Ohlmann P, Rha SW, et al (2006) Correlates and Long-term outcomes of angiographically proven stent thrombosis with sirolimus- and paclitaxel-eluting stents. Circulation 113: 1108–1113. 16490815 10.1161/CIRCULATIONAHA.105.600155

[23] Breet NJ, van Werkum JW, Bouman HJ, Kelder JC, Ruven HJ, et al (2010) Comparison of platelet function tests in predicting clinical outcome in patients undergoing coronary stent implantation. JAMA 303: 754–762. 20179285 10.1001/jama.2010.181

[24] Shaw JA, Andrianopoulos N, Duffy S, Walton AS, Clark D, et al (2008) Renal impairment is an independent predictor of adverse events post coronary intervention in patients with and without drug-eluting stents. Cardiovasc Revasc Med 9: 218–223. 18928945 10.1016/j.carrev.2008.05.002

[25] Stein B, Weintraub WS, Gebhart SP, Cohen-Bernstein CL, Grosswald R, et al (1995) Influence of diabetes mellitus on early and late outcome after percutaneous transluminal coronary angioplasty. Circulation 91: 979–989. 7850985 10.1161/01.cir.91.4.979

[26] Siller-Matula JM, Delle-Karth G, Christ G, Neunteufl T, Maurer G, et al.(2012) Dual non-responsiveness to antiplatelet treatment is a stronger predictor of cardiac adverse events than isolated non-responsiveness to clopidogrel or aspirin. Int J Cardiol. *In press*. 10.1016/j.ijcard.2012.01.016 22305813

[27] van Werkum JW, Heestermans AA, Zomer AC, Kelder JC, Suttorp MJ, et al (2009) Predictors of coronary stent thrombosis. J Am Coll Cardiol 53: 1399–1409. 19371823 10.1016/j.jacc.2008.12.055

[28] Lee SW, Park SW, Yun SC, Kim YH, Park DW, et al (2010) Triple antiplatelet therapy reduces ischemic events after drug-eluting stent implantation: Drug-Eluting stenting followed by Cilostazol treatment REduces Adverse Serious cardiac Events (DECREASE registry). Am Heart J 159: 284–291. 20152228 10.1016/j.ahj.2009.11.014

